# Supplementary figures and images for: Reduced HLA-I Transcript Levels and Increased Abundance of a CD56dim NK Cell Signature Are Associated with Improved Survival in Lower-Grade Gliomas
Source: Cancers (Basel). 2025 May 5;17(9):1570. doi: 10.3390/cancers17091570 (PMC12071263; doi:10.3390/cancers17091570)

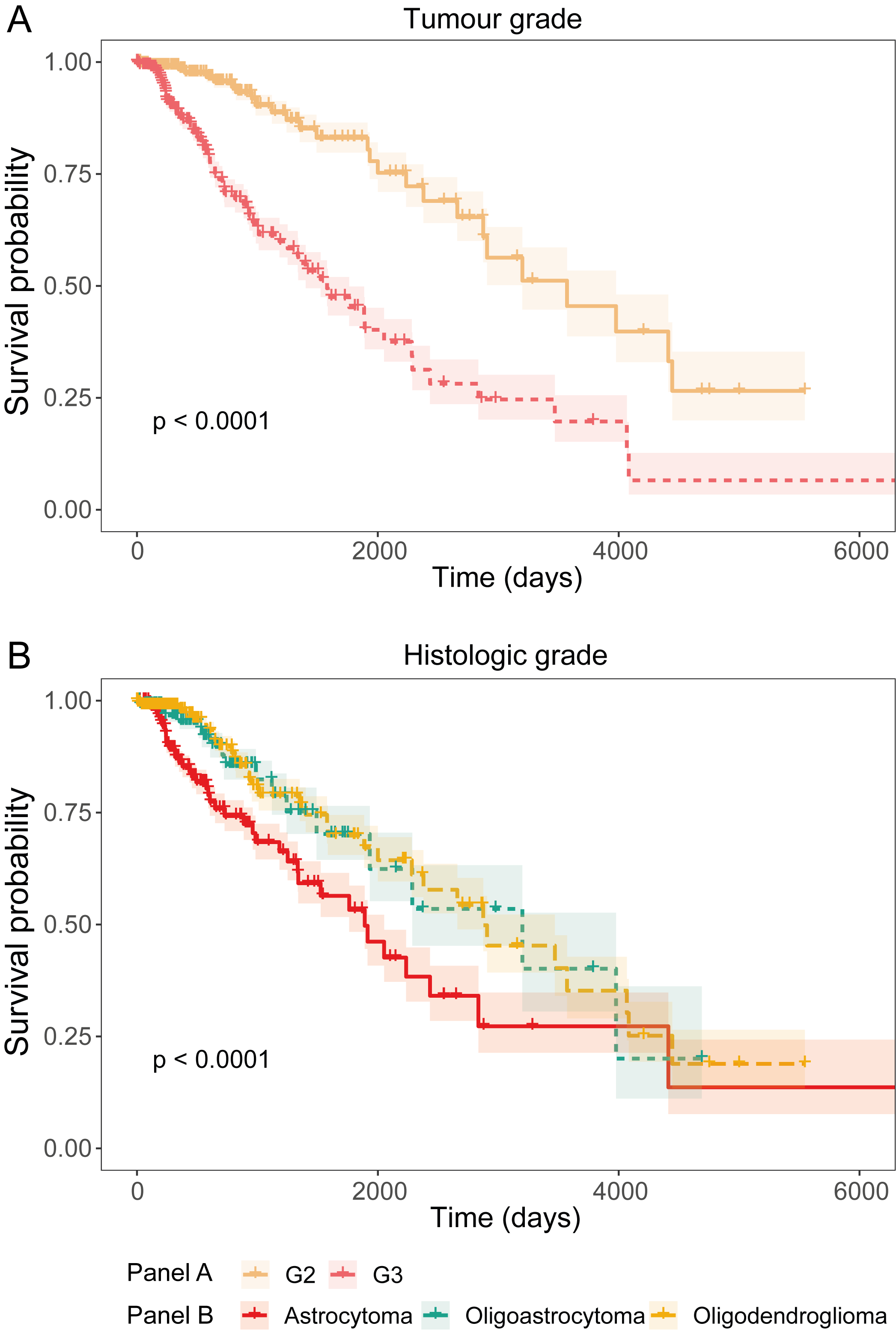

Supplement: Supplementary file 1 [file cancers-17-01570-s001.zip › cancers-3525426-supplementary/Supplementary Figure 1.tif]

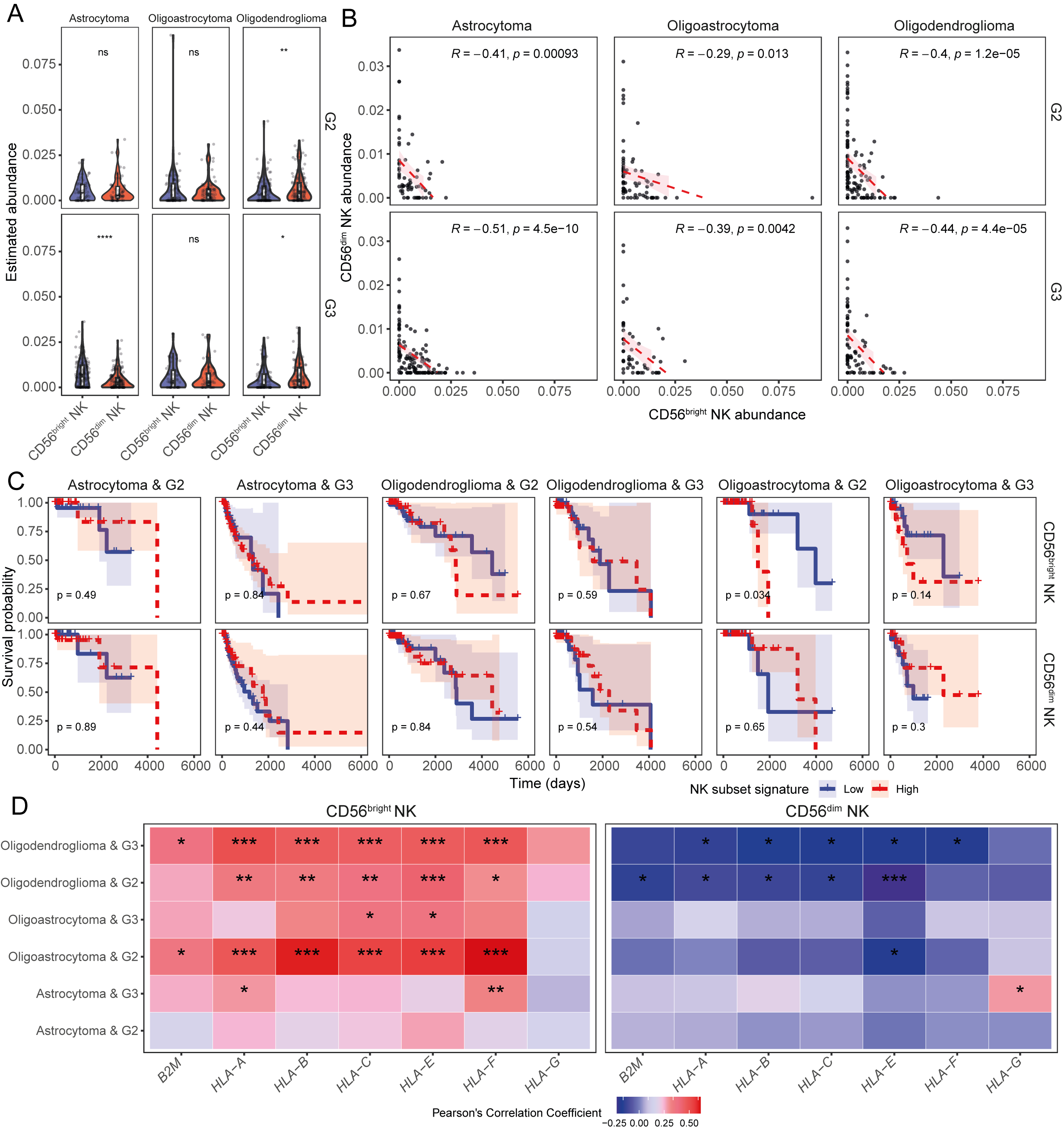

Supplement: Supplementary file 1 [file cancers-17-01570-s001.zip › cancers-3525426-supplementary/Supplementary Figure 2.tif]

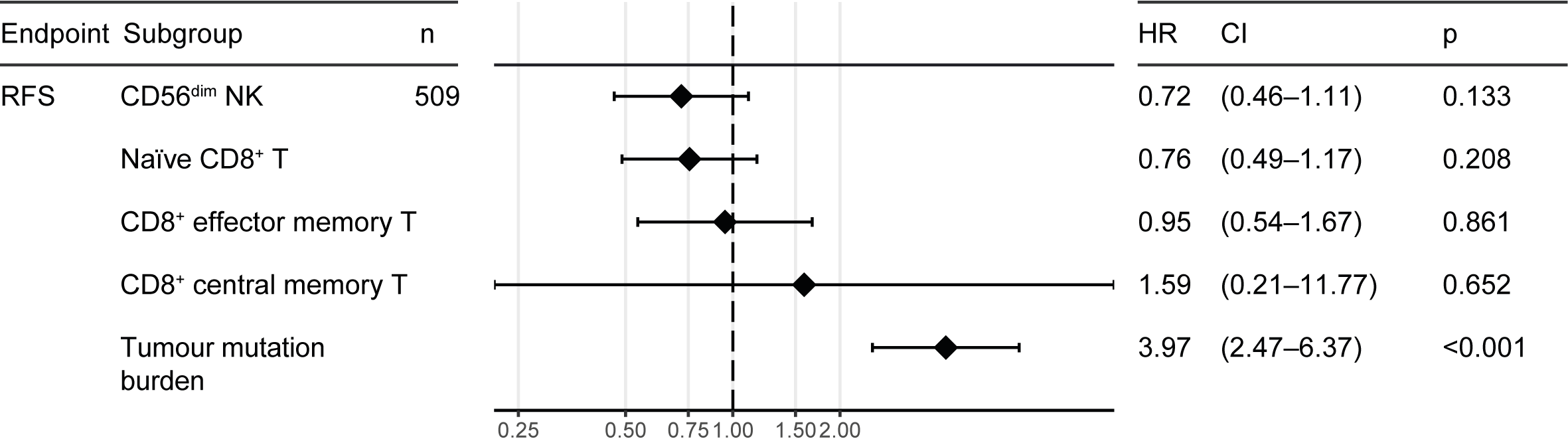

Supplement: Supplementary file 1 [file cancers-17-01570-s001.zip › cancers-3525426-supplementary/Supplementary Figure 3.tif]

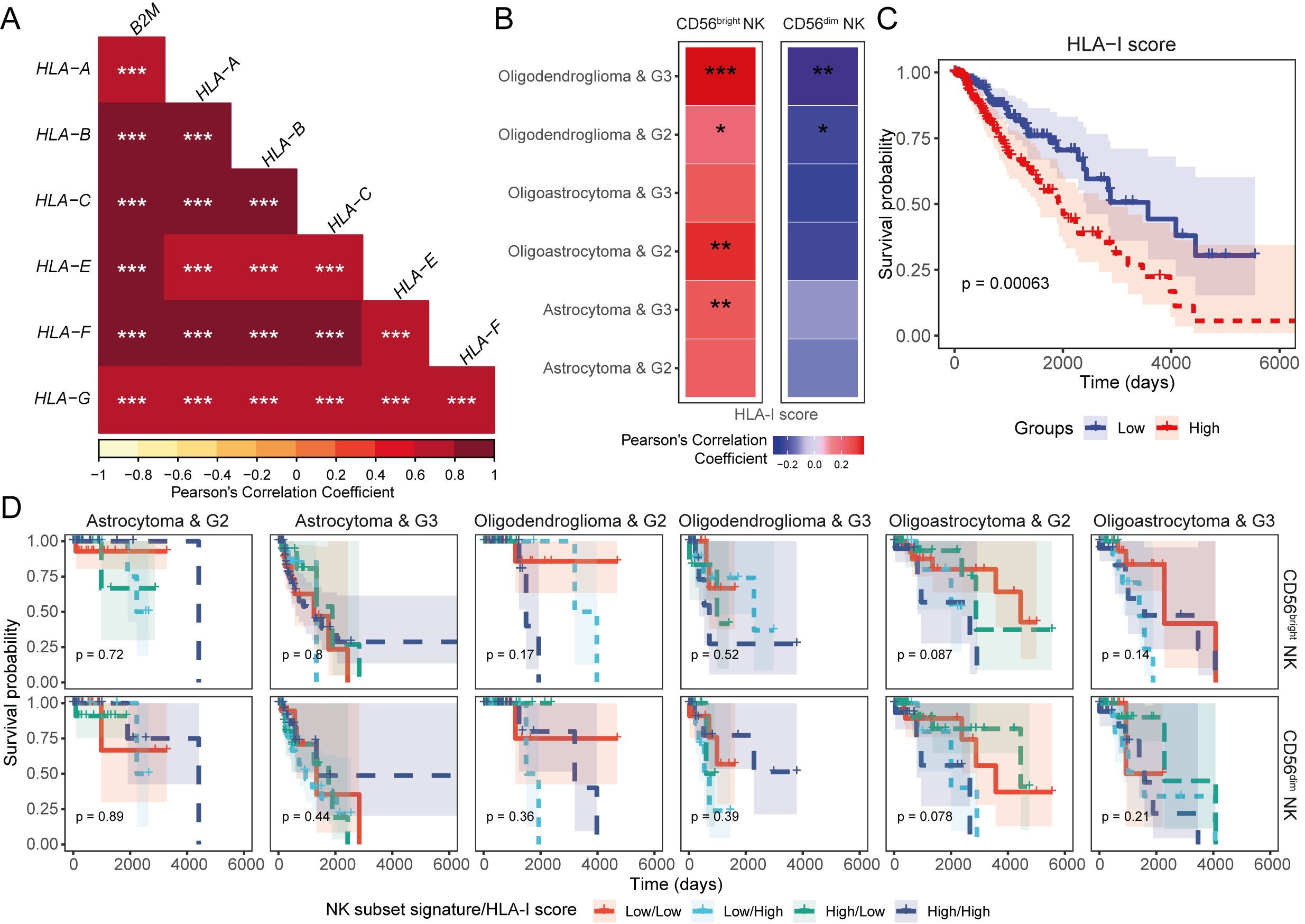

Supplement: Supplementary file 1 [file cancers-17-01570-s001.zip › cancers-3525426-supplementary/Supplementary Figure 4.tif]

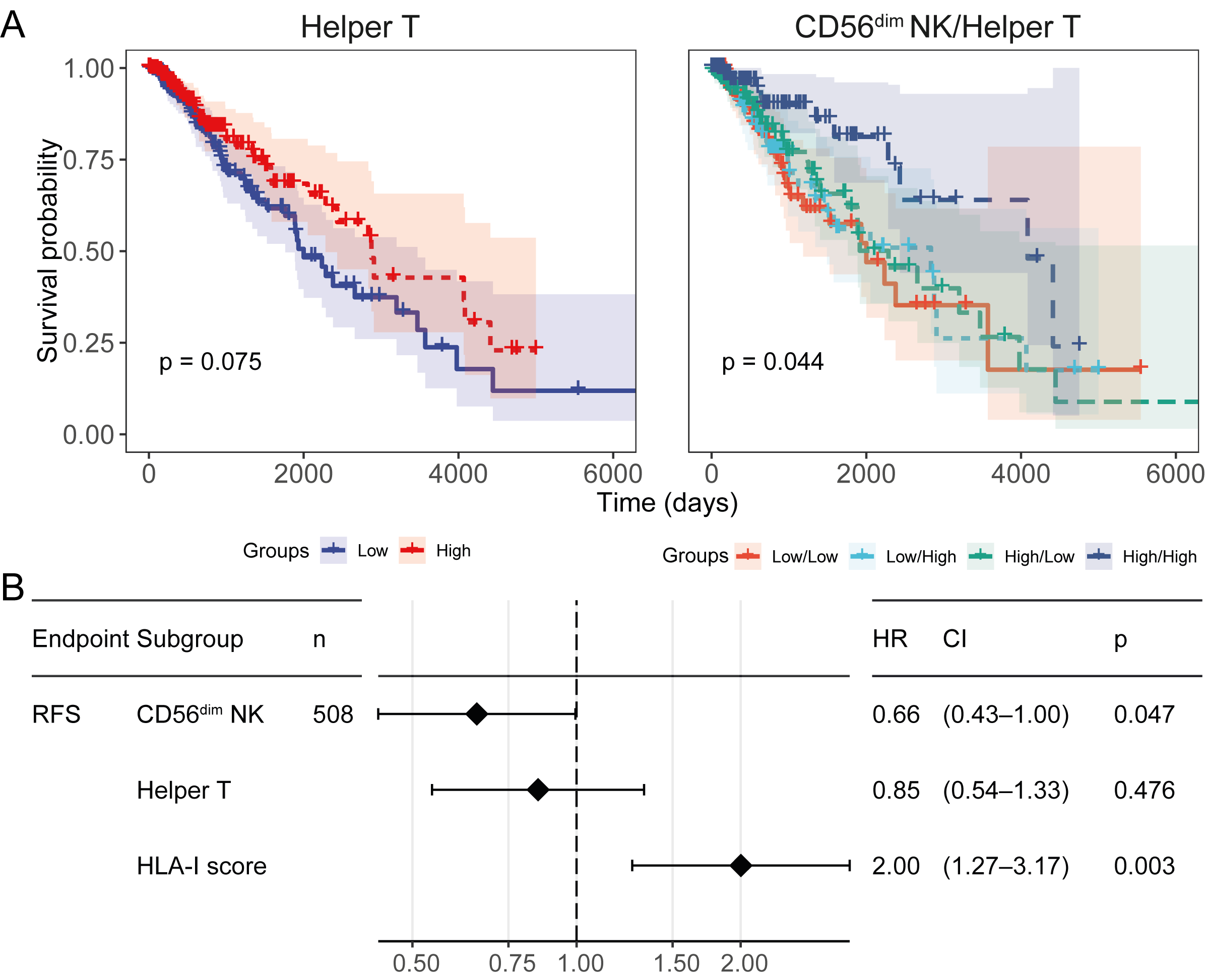

Supplement: Supplementary file 1 [file cancers-17-01570-s001.zip › cancers-3525426-supplementary/Supplementary Figure 5.tif]

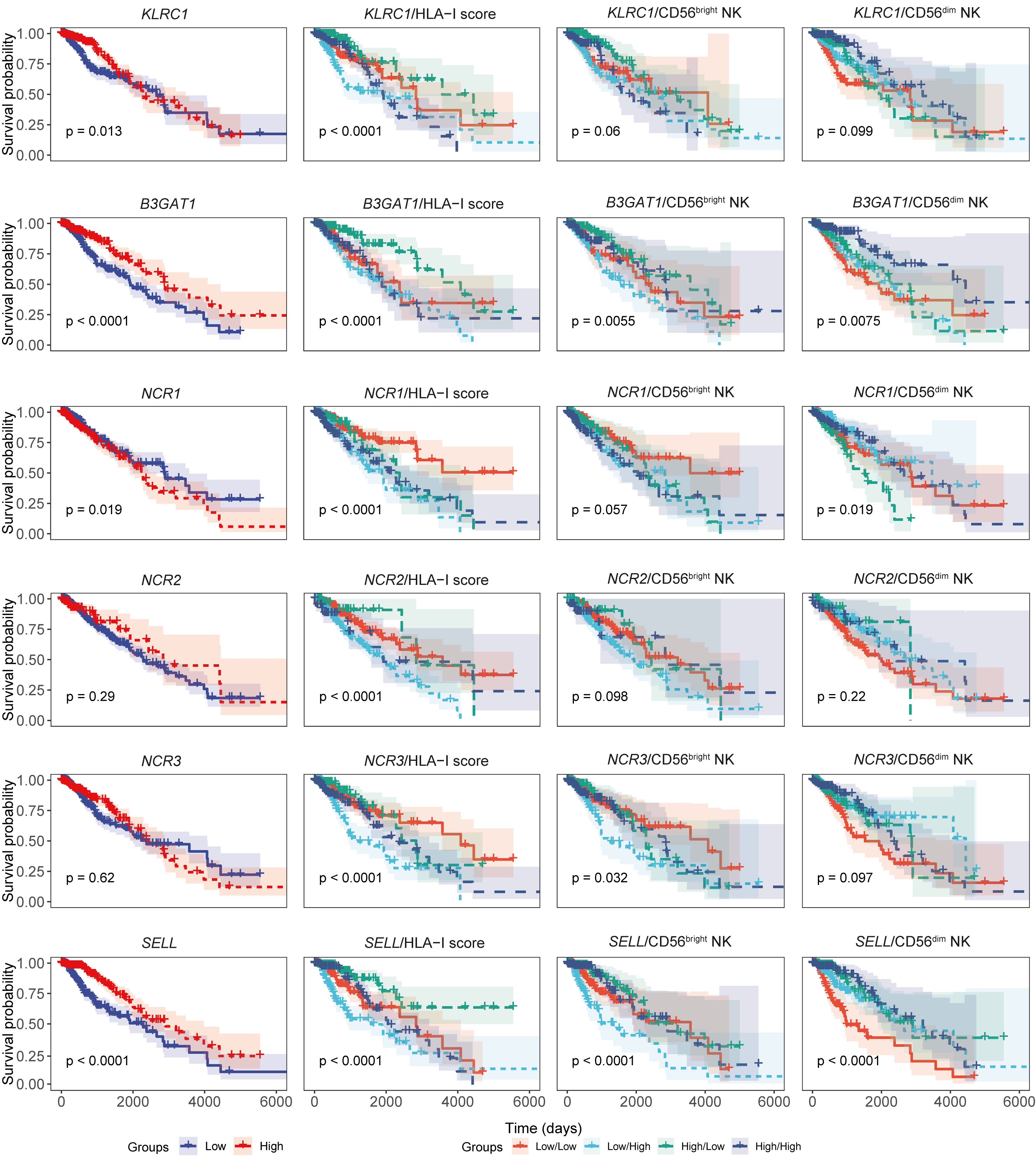

Supplement: Supplementary file 1 [file cancers-17-01570-s001.zip › cancers-3525426-supplementary/Supplementary Figure 6.tif]

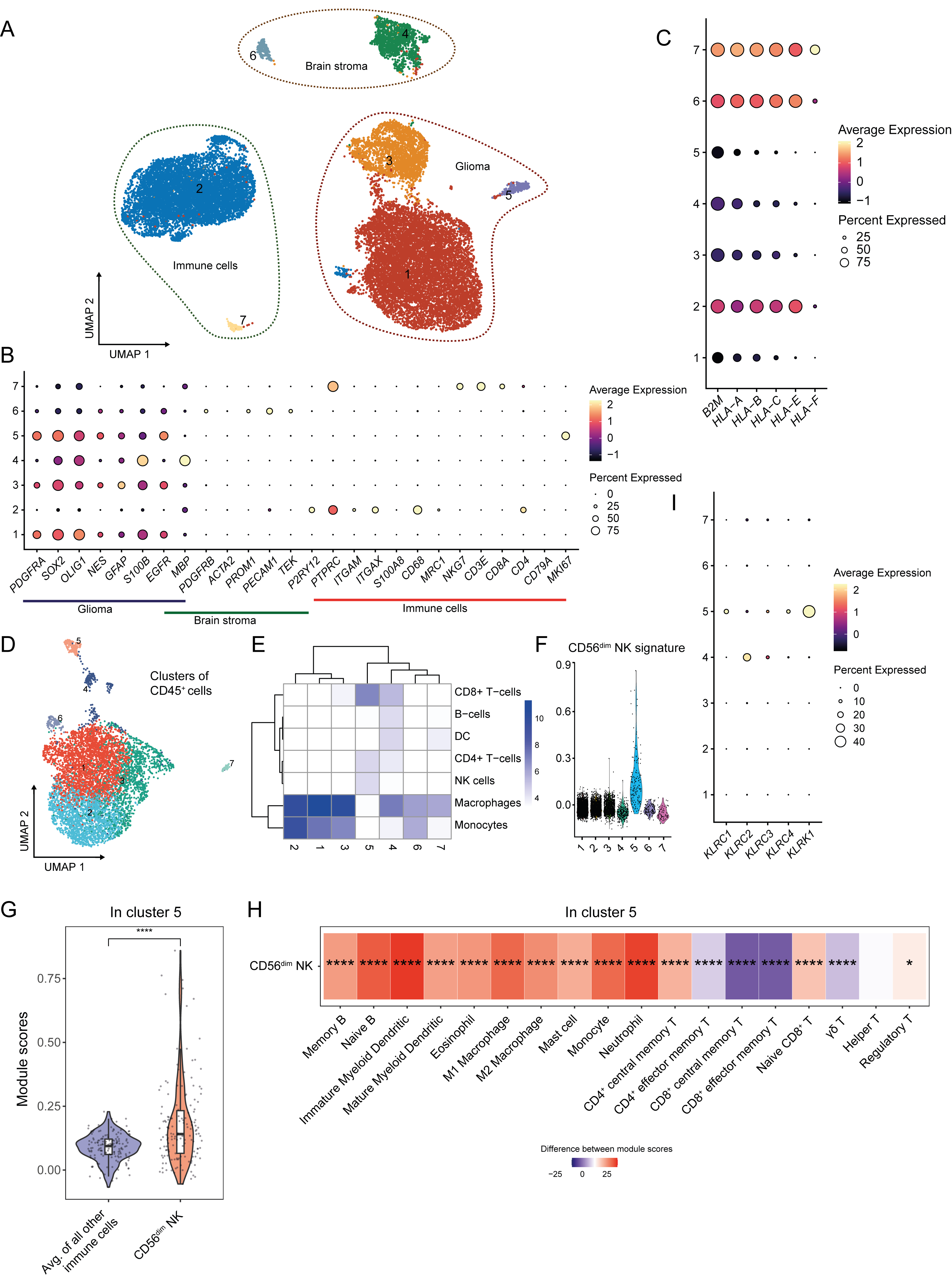

Supplement: Supplementary file 1 [file cancers-17-01570-s001.zip › cancers-3525426-supplementary/Supplementary Figure 7.tif]

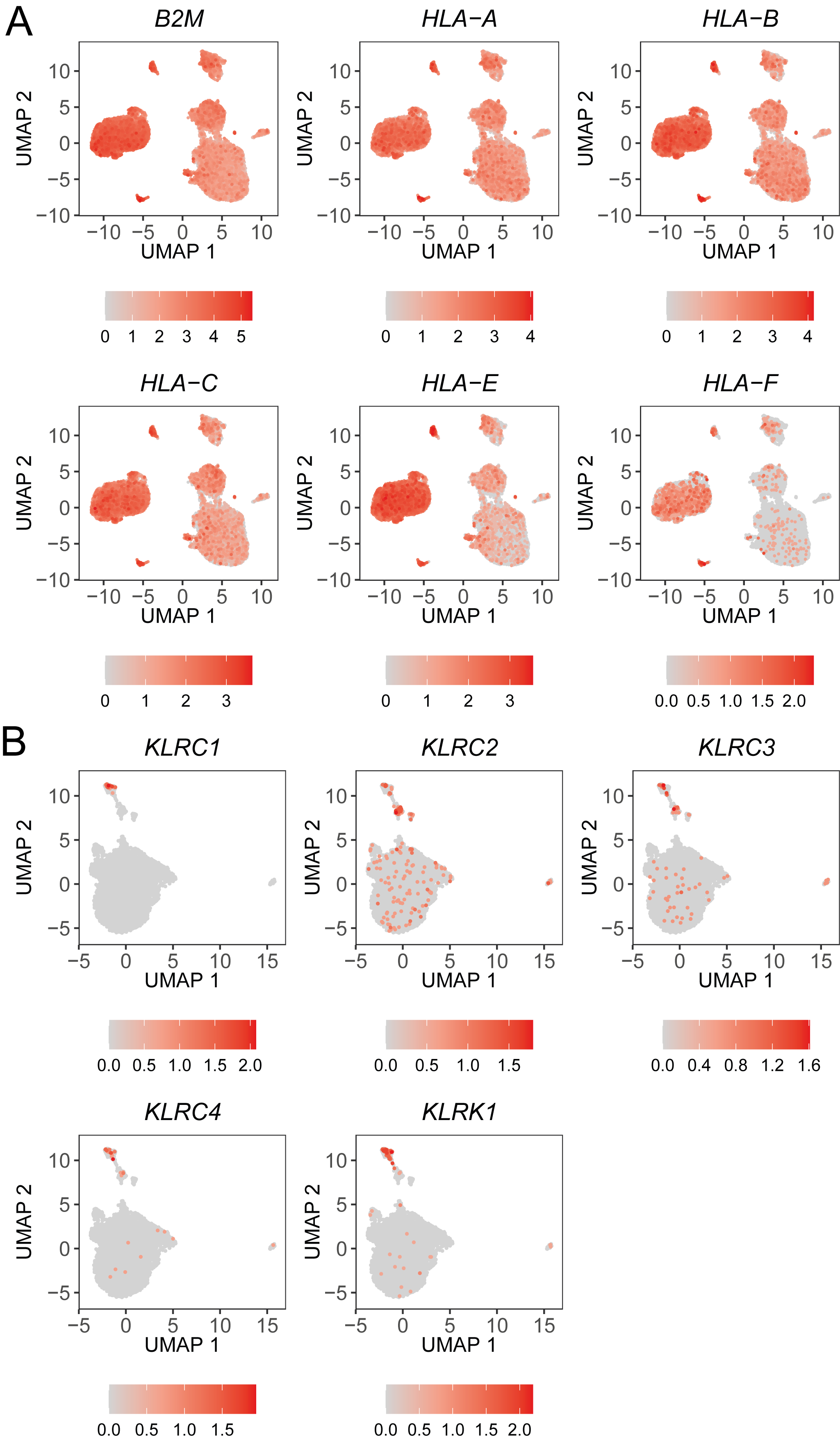

Supplement: Supplementary file 1 [file cancers-17-01570-s001.zip › cancers-3525426-supplementary/Supplementary Figure 8.tif]

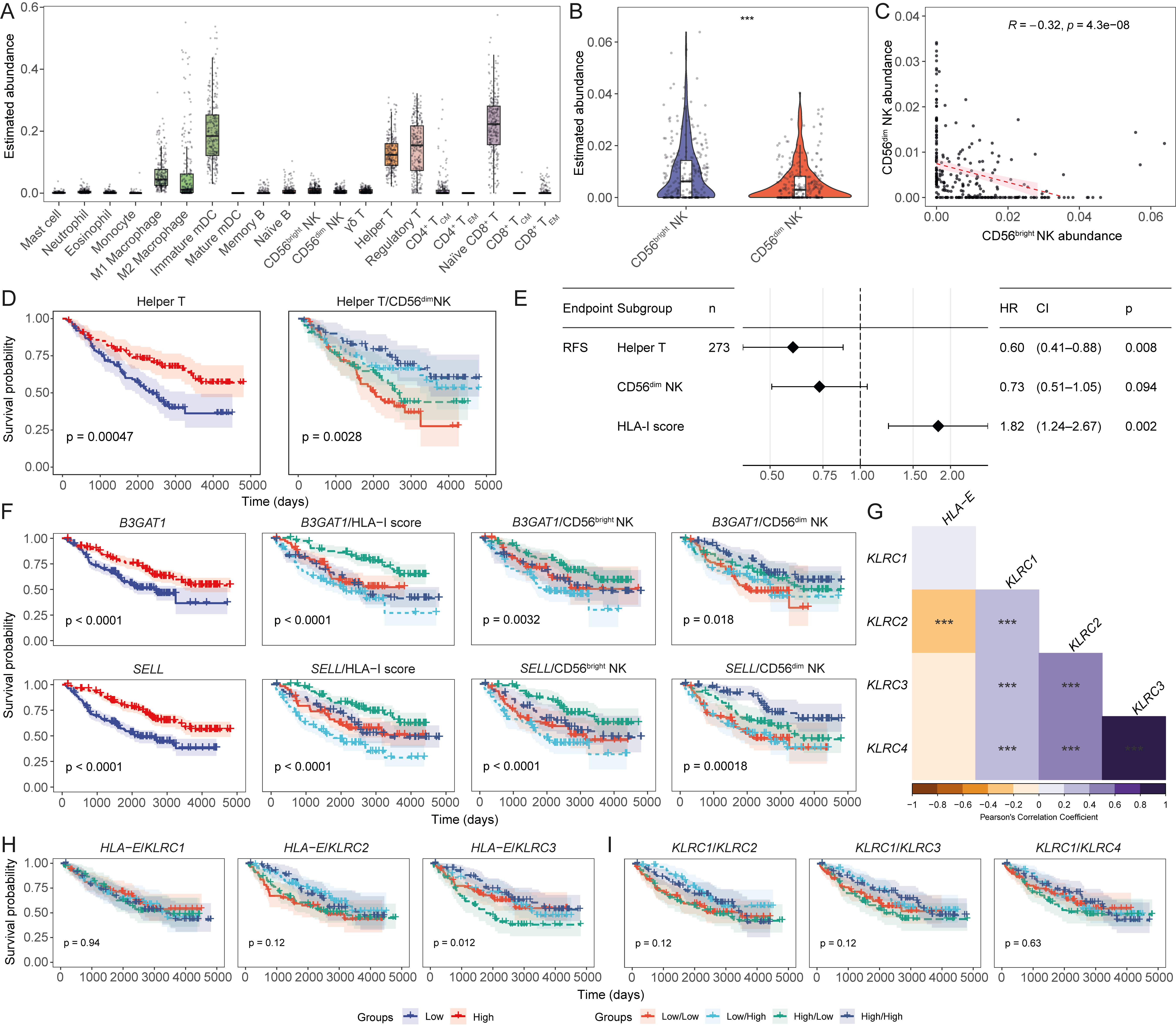

Supplement: Supplementary file 1 [file cancers-17-01570-s001.zip › cancers-3525426-supplementary/Supplementary Figure 9.tif]
